# Cognitive penetrability and emotion recognition in human facial expressions

**DOI:** 10.3389/fpsyg.2015.00828

**Published:** 2015-06-19

**Authors:** Francesco Marchi, Albert Newen

**Affiliations:** Department of Philosophy II, Ruhr University Bochum, Bochum, Germany

**Keywords:** cognitive penetrability, emotion recognition, adaptation, facial expressions, social perception

## Abstract

Do our background beliefs, desires, and mental images influence our perceptual experience of the emotions of others? In this paper, we will address the possibility of cognitive penetration (CP) of perceptual experience in the domain of social cognition. In particular, we focus on emotion recognition based on the visual experience of facial expressions. After introducing the current debate on CP, we review examples of perceptual adaptation for facial expressions of emotion. This evidence supports the idea that facial expressions are perceptually processed *as wholes*. That is, the perceptual system integrates lower-level facial features, such as eyebrow orientation, mouth angle etc., into facial compounds. We then present additional experimental evidence showing that in some cases, emotion recognition on the basis of facial expression is sensitive to and modified by the background knowledge of the subject. We argue that such sensitivity is best explained as a difference in the visual experience of the facial expression, not just as a modification of the judgment based on this experience. The difference in experience is characterized as the result of the interference of background knowledge with the perceptual integration process for faces. Thus, according to the best explanation, we have to accept CP in some cases of emotion recognition. Finally, we discuss a recently proposed mechanism for CP in the face-based recognition of emotion.

## Introduction: What is Cognitive Penetration? Does it Really Occur?

Cognitive penetrability is a phenomenon that occurs if higher-level cognitive states, such as beliefs, desires, intentions, etc., can directly influence perceptual experience. In other words, if cognitive penetration (CP) takes place, what one, believes, desires, intends, etc., may alter what one sees, hears, etc. It is currently a matter of debate whether such a phenomenon occurs and, if it does, under which circumstances it is to be expected and how it is to be characterized. A definition of CP is offered by Stokes (2013):

(CP) A perceptual experience E is cognitively penetrated if and only if (1) E is causally dependent on some cognitive state C and (2) the causal link between E and C is internal and mental. (Stokes, 2013, p. 650)

We shall adopt this definition as a starting point. The main advantage of the definition is that by emphasizing that the relevant link between C and E must be *internal and mental*, it clearly excludes instances of bodily movement and changes in non-mental bodily states from the domain of CP. In this section, we introduce the current debate on CP and review some of the reasons for thinking that such a phenomenon occurs, before exploring its possible consequences for the realm of social cognition.

In the twentieth century, the possibility of CP was the core idea behind the *new look* movement in psychology, which studied several alleged cases, albeit without appeal to the precise notion of CP (Bruner and Goodman, 1947; Bruner and Postman, 1949). Later, the idea was almost abandoned in the light of severe criticisms from Fodor (1984, 1988) and Pylyshyn (1984, 1999), who were concerned with the characterization of a reliable visual system that is capable of representing the world adequately, i.e., of delivering some true information. Fodor (1984, 1988) and Pylyshyn (1984, 1999), who introduced the current terminology of penetrability, think of vision as a serial bottom-up process that, roughly, encompasses stimulus onset to categorization. Accordingly, they present several arguments against the possibility of CP. One famous example is Fodor’s argument about the impenetrability of visual illusions such as the Müller-Lyer illusion (see below). Driven by the consideration that in order to function quickly and reliably, part of the visual system must work independently of any other cognitive subsystem and domain, Pylyshyn (1999) describes a functionally characterized early visual system that he calls *early vision* (EV), and he reviews several forms of psychological evidence motivating the proposed move. Raftopoulos (2014) has argued for EV on neurophysiological grounds, offering a temporal characterization of EV as the first 100 ms of visual processing. He is led by the observation that there is as yet very little evidence of any top-down modulation of the visual system from areas higher in the brain’s cortical hierarchy during this time period. Hence, according to Fodor, Pylyshyn, and Raftopoulos, a significant part of the visual system, and, by extension, its counterparts in other sensory modalities must be considered to be modular in a strong sense. Part of the visual system is domain-specific, an inborn system that can only be influenced by inner-sensory information. It follows from this last point that the processes of the primary visual system cannot be influenced by non-perceptual information. This is especially the case with regard to higher-level cognitive information like background beliefs or mental images. This is the core idea of cognitive impenetrability.

As previously mentioned, one central observation offered in support of the impenetrability thesis is the Müller-Lyer illusion: even if we know that the two arrows have the same length, we continue to perceive one as being shorter than the other. Our perceptual experience seems to be impenetrable to our knowledge of the line’s length. However, some researchers have recently challenged the impenetrability claim, observing that in some cultures the illusion does not arise (MacCauley and Henrich, 2006). How can we account for this? One could describe this as a case of long-term adaptation, or of perceptual learning effects that remain intra-perceptual. But how could this modification of perceptual processing take place? The reasoning behind the objection to the impenetrability argument is, roughly, that people who live in highly carpentered environments may develop a form of implicit perceptual knowledge about edges, corners, and relative distances of geometrical displays that determine the illusion, since the phenomenon is not observed (or is observed to a lesser degree) in cultures that live in non-carpentered environments. Such implicit knowledge is connected to development and long term perceptual interaction between subjects and their environment and, as such, may be relatively stable and not easily overwritten by the currently entertained belief that the two lines are equal. However, if it is indeed a form of knowledge that determines the illusion, under certain assumptions the Müller-Lyer case can be considered evidence of long-term (diachronic) CP.

Pylyshyn also allows for two kinds of interactions between perception and cognition that are compatible with his impenetrability claim. Specifically, higher-level cognitive information may either influence attention, thereby modifying the input to the visual system, or modify the output of the primary visual system after EV has done its work. Both alternatives leave EV impenetrable. Pylyshyn writes:

“Our hypothesis is that cognition intervenes in determining the nature of perception at only two loci. In other words, the influence of cognition upon vision is constrained in how and where it can operate. These two loci are: (a) in the allocation of attention to certain locations or certain properties prior to the operation of early vision […] (b) in the decisions involved in recognizing and identifying patterns after the operation of early vision. Such a stage may (or in some cases must) access background knowledge as it pertains to the interpretation of a particular stimulus.” (Pylyshyn, 1999, p. 344)

Therefore, in arguing against the impenetrability view, the principal challenge is to present convincing cases where the influence cannot be explained with reference to either of the strategies proposed by Pylyshyn, and to show that cognitive information modifies the primary visual system.

In the last decade, there has been a substantial increase in the literature describing in detail those aspects of brain architecture that are compatible with CP. Hard-wired bottom-up mechanisms are not found in the brain: perception is much more interactive and far-reaching in several respects: (i) concerning connectivity, there are many more feedback connections from higher cognitive areas to the primary visual cortex than feedforward connections to higher cognitive areas (e.g., Salin and Bullier, 1995); (ii) concerning timing, there is evidence to suggest that the timing allows for an early activation of brain areas that, if the bottom-up processing view were correct, should only be activated later. The time course of visual processes in V1 and V2 is such that we cannot presuppose simple serial feedforward processing. For example, in the processing of images eliciting perception of illusory contours, the activation of V1 caused by illusory contours emerges 100 ms after stimulus onset in the superficial layers of V1, and at around 120–190 ms in the deep layers of V1. However, in V2, the illusory contour response begins earlier, occurring at 70 ms in the superficial layers and at 95 ms in the deep layers (Lee and Nguyen, 2001). Thus, we must presuppose an interactive temporal dynamics. Furthermore, Bar (2003, 2009) argues that the prefrontal cortex can be activated very early in the processing of a stimulus and its context, and that it can interact top-down with the visual processing of that stimulus before its completion. Thus, a purely bottom-up view of visual processing is not correct if we adhere to classical views about visual areas like V1 to V5, and nor can such a view adequately account for the relation between ventral visual areas and the prefrontal cortex. We will come back to this issue when speculating about the mechanism of CP. The available evidence so far indicates that CP is a physiological possibility. Having established that CP is physiologically possible, we will now present evidence from empirical studies that cannot be adequately explained without relying on CP.

## Core Examples in the Debate

Macpherson (2012) reviews an experiment (Levin and Banaji, 2006)^1^ in which knowledge and expectations about the race and skin color of human faces biases a perceptual color-matching task. There are four versions of the experiment. We cannot present all of these in detail (but see Macpherson, 2012; Stokes and Bergeron, 2015, for further discussion). For present purposes, we will focus on the second version, which we take to be the least controversial. Subjects had to adjust a uniform patch of gray to the color of a computer generated target face, which was averaged to display ambiguous facial traits between prototypical African-American and Caucasian faces. The ambiguous face was presented next to either a prototypical African-American or Caucasian face, and all the stimuli were adjusted to the exact same color (surface lightness). The African-American faces and the Caucasian faces were labeled, respectively, “BLACK” and “WHITE,” while the ambiguous face was labeled either “BLACK” or “WHITE,” depending upon whether it was presented next to the Caucasian or African-American face respectively. The experimenters found that even when subjects were presented with the same target stimulus, namely the ambiguous face, they adjusted the patch of gray to a darker shade when it was labeled “BLACK” and to a lighter shade when labeled “WHITE.” The take of the experimenters on this result was that the subject’s knowledge and expectations about the skin-tone associated with a certain race, as triggered by the label, altered their perceptual experience of the color of the target ambiguous face. This experiment has the advantage of requiring the subjects to perform an on-line perceptual-matching task, i.e., the results are not based on subjects’ reports or introspections. This methodology aims to rule out several alternative explanations to CP, such as cognitive influences on the subject’s post-perceptual judgments or pre-perceptual attentional shifts.

Stokes (2014) reports an experiment with a very similar methodology performed by Witzel et al. (2011). Experimenters found that when strongly color-biasing shapes (e.g., a Smurf or a Coca-Cola icon) were presented in a random target-color and had to be adjusted for color to match a gray background, subjects chose a matching shade of gray in the opposite hue-range to the thematic-color. To give an example, a subject may adjust a randomly colored Smurf slightly in the yellow hue, which is the opposite hue to the thematic color of the smurf (blue). This result is to be expected if the subject sees the randomly colored Smurf as *bluish*. Such an effect did not occur in the control condition, where the same procedure was applied when color-neutral shapes (e.g., a sock or a golf ball) were presented. Importantly, subjects in the experimental condition did not choose a shade of gray in the opposite hue-range to the random target-color of the biasing shapes, but in that of the thematic-color (the usual color of that object). Accordingly, experimenters concluded that the subjects’ knowledge of the thematic color slightly altered their perceptual experience of the target-color. Such results provide support for the idea that CP actually occurs in color perception. However, as Stokes rightly points out, the literature in this field is in its infancy, and few experiments have employed the methodology of on-line perceptual matching. It is plausible that as the literature develops, more evidence for CP in different domains of perceptual experience will emerge. Further evidence of CP includes the evaluation of steepness of slopes (Bhalla and Proffitt, 1999; Durgin et al., 2009) and spatial perception (Stefanucci and Geuss, 2009)^2^.

Another experiment demonstrating the online-influence of concepts on perception was carried out by Winawer et al. (2007). They presented Russian and English speakers with color samples of different shades of blue. The experiment was based on different ways of categorizing shades of “blue” in the two languages: Russian speakers lexicalize the “blue” category by means of two basic level terms: “siniy” for darker blues and “goluboy” for lighter blues. In contrast, English speakers have just one basic-level term (“blue”). The students were asked to decide as quickly as possible whether a color presented at the top matched a color on its left or its right exactly. While all the shades presented were in the same category of “blue” for English speakers, the colors fell under two different basic categories for the Russians. Winawer et al. (2007) found that the Russians—but not the English—had slower reaction times (RTs) in same-color trials (comparing a darker and a lighter shade of blue) than in between-colors trials (comparing a light blue and green).

In addition to the RT results presented above, Carruthers (2015) reviews an analog experiment (Mo et al., 2011) done using EEG-data. The experiment relies on mismatch negativity, measured after 150 ms, indicating the online-influence of early visual processes. Mo et al. (2011) reported mismatch negativity in native speakers of Mandarin, who distinguish two shades of green but not of blue:

“Subjects were required to fixate on a central cross flanked by two colored squares, and were asked to respond as swiftly as possible whenever the cross changed to a circle. The squares were positioned so that the one on the left would be represented initially in the right hemisphere whereas the one on the right would be represented initially in the left (linguistic) hemisphere. As expected, both hemispheres showed a mismatch negativity response to changes in the presented color. But in the right hemisphere there was no difference in the amplitude of the response to changes of color within a category (one shade of green changed to another shade of green) versus across categories (a shade of green changed to a shade of blue). However, in the left (linguistic/conceptual) hemisphere there was a significant difference, with a much larger effect for cross-category changes.” (Report taken from Carruthers, 2015)

Finally, Lupyan (2012, 2015) provides further evidence that this experiment cannot be interpreted as involving modular processing of the primary visual cortex. In addition, he offers an alternative model of how inferential processes produce online-modifications in perceptual experience, and provides further examples of CP that are especially related to the interactions between perception and language processing.

Thus, given the available evidence, which does not involve core dimensions of social cognition (except for the aspect of race), it is plausible to accept CP, in principle, for cases of object perception and color perception. But what about social perception? Can we plausibly extend the discussion of CP to this area? In this paper, we aim to show that cognitive penetrability also shapes our perception of socially relevant information. We focus on a clear case of perceptual recognition of socially relevant information, and, specifically, on face-based recognition of basic emotions.

Before proceeding further, however, we must point out that the claim that our perceptual experience of another person’s emotion (the “emotion-percept”) is influenced by memorized images or background beliefs is not entirely new. One line of argument, mainly inspired by phenomenology, supports the idea of CP of emotion recognition by arguing that recognizing the emotions of others is primarily a *direct perceptual* achievement (Gallagher, 2008; Zahavi, 2011; Krueger, 2012; Stout, 2012). Although we sympathize with the direct perception claim with respect to basic emotion recognition (see below and Newen et al., 2015), we want to develop our argument in this article in such a way as to be acceptable even for those who deny direct perception. If we cannot presuppose that the content of a percept is rich, i.e., that it involves rich images as well as conceptual information, it becomes much more difficult to argue that obvious changes in the recognition of emotion rely on a change of the percept, instead of a change of judgment alone. Furthermore, our main claim converges with the position that emotion, cognition and perception cannot be neatly separated into distinct modules (Pessoa, 2013; Colombetti, 2014), which draws support from emotion science. But it is important to note that the debate about CP would be empty if one were to hold the view that cognition and perception could not be separated at all. Thus, we are presupposing a minimally clear separation of the *perceptual experience* (be it conceptual or non-conceptual), and the *judgment* based on this perceptual experience.

## Perceptual Adaptation and the Experience of Facial Expressions

Given the complex nature and extreme relevance of human faces in our perceptual life, it is an interesting question whether recognition of an emotion in a human face is achieved through a judgment made on the basis of perceptual experience, a purely perceptual automatic process, or an interaction between both that admits some degrees of CP. In order to argue for the third of these options, we start with the question of whether we can perceive facial expressions as wholes, or whether the evaluation of a facial expression depends on post-perceptual processes.

The structure of our argument, presented in more detail, runs as follows: in the first step, we argue for a process of feature integration in the case of facial expressions of emotions, and claim that this is a perceptual process. The integration process we have in mind consists in the gradual combination of facial features and cues into complex compounds. By discussing perceptual adaptation to facial expressions of emotions, we show that there are reasons to think that the resulting compounds, i.e., whole facial expressions, have to be considered as perceptual states. Secondly, we argue that such perceptual integration processes can be influenced by contextual background knowledge, such that we have to accept that the social perception of emotion involves CP.

Human faces are complex stimuli. They are arguably one of the richest and most reliable sources of information available to us in our everyday lives. Two of many examples of phenomena based on face perception that constitute a significant subset of the perceptual development of a healthy human subject include gaze following and joint attention. According to some researchers (Dunbar, 1998; Adams and Kveraga, 2015), the enormous amount of information conveyed by human faces is of such relevance for behavior and social interaction that it is plausible to think that humans have evolved a dedicated perceptual sub-system for quickly integrating the various social cues conveyed by a face into meaningful compounds.

The phenomenon of pareidolia (e.g., Hadjikhani et al., 2009) provides further evidence for the existence and relevance of an integration mechanism for faces: we tend, for instance, to see faces in natural collections of sand or in cloud formations, because the integrated patterns are extremely important for humans and can be easily activated in various situations. Furthermore, the widely accepted empirical model of face-based recognition of emotion proposed by Haxby et al. (2000) and Haxby and Gobbini (2011) involves the following two-step process: (1) the construction of facial identity and (2) the recognition of facial expressions. The latter, extended part of recognizing a facial expression, is supposed to involve such an integration process of core facial features. Furthermore, recent models analyze normal object perception as involving Bayesian processes of cue integration and cue combination (Ernst and Bülthoff, 2004), and it is very plausible that the principles of perception remain the same in the case of non-social objects and in the case of perceiving emotions in faces (Newen et al., 2015). Thus, it is very plausible to accept a feature integration process in the case of recognizing the expression of an emotion in a face, or recognizing a face in certain perceptual configurations. However, it is not clear whether faces and facial expressions as wholes are perceptually processed or not. In fact, it may be that even if there is a feature integration process at play, facial expressions are only recognized post-perceptually on the basis of certain perceptual arrays of lower-level features. Why should we take this integration process and its results to be perceptual?

In the present section, we present the first step of our argument as outlined above. In particular, relying on evidence recently reviewed in Block (2014), we show that in some cases the proposed result of the integration process, i.e., a whole facial expression, shows perceptual adaptation. Under the assumption that adaptation is a perceptual process, and that only perceptual states/contents may adapt, it follows that since facial expressions as wholes show adaptation, facial expressions as wholes are perceptually processed. In the next section, we show that the perceptual integration process of facial expressions may be influenced by contextual background knowledge and the subject’s beliefs.

Perceptual adaptation consists in a process where being exposed to a certain perceptual feature (or set of features), either repeatedly or for a long time, makes that feature less likely to be detected in other stimuli. One explanation for this adaptation is that the firing threshold of the neurons that code such feature in the perceptual system is raised by prolonged exposure. Block (2014)^3^ addresses this phenomenon in the case of facial expressions of emotion, focusing on the problem of whether the nature of certain adaptation effects is perceptual or cognitive.^4^ Block reviews an experiment by Butler et al. (2008). In this study, experimenters found that whether a still picture of a face displaying an emotional expression, ambiguous between anger and fear, was more or less likely to be perceived as expressing anger or fear depending upon previous exposure to a clearly fearful or clearly angry face. Most importantly, the effect was found to persist when the low-level features of the face were varied, as long as the emotion expressed was kept constant. This seems to be a clear case of perceptual adaptation. The exposure to a clearly angry face raises the threshold for detecting anger-related features in the subsequently presented ambiguous face, and the opposite happens in the case of exposure to a clearly fearful face, which is then perceived as expressing fear.

Concerning this case, Block writes:

“[…] can we be sure from introspection that those “looks”- [fearful/angry] - are really perceptual, as opposed to primarily the “cognitive phenomenology” of a conceptual overlay on perception, that is, partly or wholly a matter of a conscious episode of perceptual judgment rather than pure perception?” (Block, 2014, p. 7)

Providing an answer to this question is difficult, but Block thinks that there is reason to reply in the affirmative, and thus to consider adaptation to facial expression to be a perceptual phenomenon. The preliminary reason for this conclusion, according to Block, is that concepts are in general much more resilient to adaptation than percepts. In particular, Block argues that in cases of ambiguous pictures, we find a form of multi-stable perception in which two percepts are alternatively perceived, and that this switching is the result of perceptual adaptation.

The alternation of the two percepts works according to the three properties of exclusivity (only one percept at a time), inevitability (the alternation will surely happen at some point), and randomness (there is no function of duration for each percept). Block assumes that correspondent judgments and beliefs are not subject to an alternation that works according to the same three properties even in highly conceptually ambiguous situations, and concludes that there is no such thing as conceptual adaptation.^5^

As further evidence, Block considers an experiment by Schwiedrzik et al. (2014), in which subjects were first exposed to a clearly oriented (either 90°or 0°) grid-like stimulus, and had to report the orientation. Afterward, they had to evaluate the direction of tilt of an ambiguously oriented grid-like stimulus of the same kind. The experimenters found that there was an adaptation effect in the reports of the orientation of the second stimulus that depended upon the *objective tilt* of the first stimulus, not its *reported tilt*. In other words, when there was a discrepancy between the objective and the reported tilt of the first stimulus, the subsequent adaptation effect was consistent with the former, not the latter. According to Block, this means that subjects showed an adaptation effect that depended exclusively on what they actually saw, not on what they thought they saw. Therefore, adaptation effects have to be considered to be purely perceptual phenomena.

For present purposes, it is very important to note that Schwiedrzik et al. (2014) investigated adaptation and the different phenomenon of priming, in the same experiment, as two opposite effects. Priming is basically the facilitation of detecting a certain perceptual feature (or set of features) as triggered by a briefly presented previous stimulus, called the prime. While adaptation is exclusively triggered by prolonged exposure to a perceptual stimulus, priming can be triggered by a prime of the same or similar perceptual kind as the target, or by a prime that is semantically related to the target, i.e., a word. Schwiedrzik et al. (2014) monitored the cortical activity of the subjects and, consistent with what has just been said, found that adaptation involved only areas V1 and V2, while priming involved a wider range of cortical areas. This data shows that adaptation is largely independent of the subject’s judgment about their experience, and that the locus of adaptation is mainly in the visual cortical areas, lending further support to the idea that adaptation must be considered a purely perceptual phenomenon.

Facial expressions of emotions are complex stimuli, constituted by specific arrangements of lower-level facial cues like eyebrow orientation, mouth shape, etc. Hence, if facial expressions of emotions as a whole show adaptation and, conversely, if a perceptual system can adapt to facial expressions as a whole, this means that such a system is capable of detecting lower-level facial features and integrating them into meaningful compounds,^6^ even before corresponding judgments about the emotion expressed by the faces are formed. If this is correct, it is clear that the integration-process we just described is sensitive to and is directly affected by different factors such as lower-level feature saliency and different kinds of attention. In addition, as we aim to show, the perceptual integration process can be influenced by previously formed expectations and beliefs. We will now present a case study in which one finds an effect that seems to be just such a case, where the integration process is influenced by contextual background knowledge.

## Face-Based Recognition of Emotion is Sensitive to Background Knowledge

The upshot of Block’s argument is that it is plausible to think that facial expressions of emotions are processed as compounds that are largely the result of a feature integration process belonging to perception, insofar as it shows adaptation. The reasons, as we have seen, are that adaptation to facial expression is at least partly independent of the lower-level features constituting the expression, and that concepts and other cognitive features do not adapt the way percepts do, thus ruling out the possibility that adaptation depends on higher-level cognitive features. What adaptation shows is that if the perceptual system is exposed over a prolonged period to a facial expression of emotion x, the exposure will affect the integration process such that it will be less likely that x is recognized as being expressed by a subsequent similar facial expression. In other words, the integration process that gives rise to the emotionally meaningful perceptual compound associated with x is sensitive to stimulus familiarity. In this section, we will present a case in which the same perceptual integration process seems to be sensitive to the background knowledge of the subject. We argue that if this is the case, then we are dealing with the clear and direct influence of knowledge on perceptual processing and, plausibly, on the corresponding perceptual experience. If this is correct, such a case qualifies as an instance of CP in social perception.

The experiment of Butler et al. (2008) reviewed by Block shows that perceptual experience of facial expressions, expressions of emotion in particular, is sensitive to adaptor stimuli that bias the interpretation toward a different emotion. Moreover, Block’s discussion points to the fact that this phenomenon may plausibly be considered purely perceptual. Our case study presents a very similar effect on the facial expression of emotion, in which different emotions are recognized as being expressed by the same face. The experimental condition is actually very similar to Butler et al. (2008). The main difference between the two studies is that in the case we report, what triggers the shift in the integration process is not a perceptual adaptor (like another facial expression, as in the above case), but a subject’s expectations, which are driven by her background knowledge and activated by a form of conceptual priming.

The experiment we will discuss was carried out by Carroll and Russell (1996). The participants had to evaluate the emotion expressed by a human face. Subjects were presented with combinations of faces and situations. The target stimuli were still photographs of posed facial expressions, selected from among the prototypical facial expressions of fear, anger, or sadness, as collected in Ekman and Friesen (1976). Such prototypical facial expressions have the peculiar characteristic of being reliably evaluated as expressing the same emotion across different subjects and cultures (Keltner et al., 2003), in cases where no additional information is available. Situations were provided in the form of short stories concerning the persons depicted in the stimuli. Such stories were designed to trigger an emotional response of fear, anger, or disgust. Subjects were first told the story, and then shown the picture. They then had to evaluate the emotion expressed by the face by choosing one of six possible emotion labels.

Carroll and Russell addressed the possibility that providing contextual information to subjects may alter which emotion is recognized as being signaled by the prototypical facial expressions. For simplicity, we present only the pairing of an anger-situation with a fearful face. The situation was provided in the form of the following story:

*This is a story of a woman who wanted to treat her sister to the most expensive, exclusive restaurant in their city. Months ahead, she made a reservation. When she and her sister arrived, they were told by the maitre that their table would be ready in 45 minutes. An hour passed, and still no table. Other groups arrived and were seated after a short wait. The woman went to the maitre and reminded him of her reservation. He said that he’d do his best. Ten minutes later, a local celebrity and his date arrived and were immediately shown to a table. Another couple arrived and were seated immediately. The woman went to the maitre, who said that all the tables were now full and that it might be another hour before anything was available.*^7^

The researchers found that when presented with such contextual information, the vast majority of subjects evaluated the face as signaling anger. When the contextual information was not presented, however, subjects evaluated the same face as expressing fear, in accordance with Ekman’s earlier findings.

Can we be sure that this effect demonstrates the influence of background knowledge on perceptual processes, and that it is not only a product of modifying our perception-based judgment?^8^ Assuming, for the reasons discussed above, that the perceptual system is capable of integrating different low-level facial cues into meaningful compounds, it is clearly possible that in the present case, the background knowledge (based on *conceptual semantic priming*) provided by the story actually interferes with such an integration process.^9^ There are two possible positions that may be taken in response to this. According to the previously mentioned approaches inspired by continental phenomenology, emotions are always directly perceptible in visual experience. If this is the case, however, the possibility that emotion recognition on the basis of facial expressions is the upshot of a cognitive inferential process of judgment [i.e., judgment shift (JS)] seems to be excluded.^10^ On the other hand, if we accept that emotion recognition may be the result of a cognitive inferential process, the question that arises is whether, under certain conditions, the perceptual experience that underlies such process may be modified by a subject’s background knowledge or some other of his cognitive states. We will not discuss the motivations for adopting either of these positions here. Instead, we will argue that even if emotions are not directly perceivable, there are reasons to think that the perceptual process that leads to emotion recognition on the basis of facial expressions is penetrated by higher-level cognitive states.

## Emotion Recognition: Perceptual Categories and Judgments

Even if one accepts *priming* in the case of the facial expression of emotions, one can still doubt that the evidence provided above constitutes a clear case of the conceptual priming of perceptual experience, as opposed to a case of the conceptual priming of perceptual judgment. We will now propose some additional reasons to support the perceptual (as opposed to conceptual) nature of the effect of background information on the recognition of emotion expressions. Our argument takes the form of an inference to the best explanation, intended to show that the CP of perceptual experience provides a better explanation for shifts in emotion attribution, as compared to the alternative explanation that involves perceptual judgments.

The phenomenon to be explained is the recognitional shift that subjects are ready to make when provided with additional information about the emotion expressed by a face, where that face is otherwise reliably taken to signal a specific emotion. Our argument takes the form of an inference to the best explanation,^11^ so we need to put two competing explanations on the table. The two alternatives we shall consider are CP and JS:

**CP**: Subjects recognize two different emotions as expressed by the same face on the basis of two different perceptual experiences of that face.

**JS**: Subjects recognize the same face as expressing two different emotions by forming two different perceptual judgments on the basis of the very same perceptual experience.^12^

There are several things to consider here. First of all, it is a widely studied phenomenon that, taken out of context, certain human facial expressions tend to signal one specific emotion and not others very reliably.^13^ Secondly, it is known that contextual information may alter the kind of emotion that the face is taken to signal. This happens both in cases of a change/enrichment of perceptual context (for the visual case, see Aviezer et al., 2008; Hassin et al., 2013) and in cases of conceptual priming, as described above. The most important point, however, is that shifts in emotion recognition do not happen arbitrarily. Even if a prototypical facial expression of fear can be taken to signal anger under certain conditions, there are some constraints that make it highly unlikely that such a prototypical expression of fear could ever be taken to signal a radically different emotion, such as joy.^14^ We shall argue that these constraints are best explained as perceptual constraints. That is to say, the different possible emotions that subjects are ready to recognize as expressed by a particular face depend on the perceptual integration of different low-level features of the face itself, like mouth shape, eyebrow orientation, gaze, and so on. We shall call such features facial cues. According to JS, a subject may recognize a prototypical facial expression of fear as expressing anger by forming different judgments on the basis of the same perceptual experience of a fearful face. If this were the case, however, we do not see how constraints on emotion recognition could be introduced in a principled way. If recognizing an emotion were only a matter of judgment, it would seem possible, regardless of the epistemic confidence of the subject, to provide enough background information for the subject to revise his judgment from one of recognizing fear to one of recognizing joy. This, as per our assumption, cannot be the case. One might argue that there are indeed such cases of radical JSs. For example, if someone were to tell you that the person in the target picture has a rare dysfunction in her facial muscles that forces her to adopt a fearful expression whenever she is joyful (and *vice versa*), you might in the end come to the correct evaluation of an expression of joy in the fearful face. This illustrates that we can adapt our judgments, but only at a later stage. We need to presuppose that—at least at the beginning of noticing such a special case—the face is rightly recognized as expressing fear and only subsequently evaluated as expressing joy, on the basis of background information. After the initially correct recognition of fear, subsequent judgments that associate the face with a different emotion can be made without constraint. But if JS were true, even the initial recognition judgment would be subject to such unconstrained flexibility, which is implausible in the light of the strong reliability of emotion recognition. Therefore, we do not see how a principled way of constraining emotion recognition can be introduced at the level of pure judgment. This is not to say that it is in general impossible to introduce such constraints, only that, as we shall see, it is much more straightforward and empirically more plausible that the required constraints work at the level of perception.

Here, one might try to reinforce JS by taking into account similarity of stimuli, and say that if we are right, then our argument should apply to a whole lot of different cases of perception-based judgment. For example, one might come up with the following case:^15^ there is a picture depicting my very similar-looking twin (but who is noticeably different in some matters of detail) wearing a red coat. If one sees the picture and knows that I like to wear red coats, one might mistakenly recognize me in the picture instead of my twin. However, the counter argument goes on, this seems to be a clear case of a mistaken perceptual judgment that requires no difference in the perceptual experience of the subject. Why cannot the case above be explained along the same lines? We argue that the consequences of such an account are less plausible than our alternative explanation. The problem that the JS explanation faces comes in the form of a dilemma. The defender of JS might either (1) propose that the two kinds of stimuli of fearful faces and angry faces are very similar to each other and (both) very different from joyful faces, or (2) claim that they are not so similar.

If one goes with (1), and proposes that such stimuli are similar, then one could say that the similarity and ambiguity between fearful and angry faces, which they do not share with joyful ones, could explain why, on the basis of the very same fearful-face experience, subjects are allowed to activate fear judgments and anger judgments but not joy judgments: so far, so good. However, in this case, one faces the serious problem of how to account for the high reliability of emotion recognition across different subjects and cultures. Even if one does not buy into the original basic-emotion framework, the studies conducted by Ekman and colleagues provide quite compelling reasons to think that the overwhelming majority of subjects^16^ are at least capable of making very clear perceptual discriminations between different facial expressions of the basic emotions: people of different cultures can reliably distinguish between anger, fear, disgust, sadness, and surprise, and can reliably combine the judgment with the facial expression, given a selection of basic emotions. How can a defender of a JS explanation account for such reliability? If some of the target faces for basic emotions of fear and anger are supposed to be very similar, we would expect a higher rate of mistakes from subjects evaluating which face expresses which emotion.

If, on the other hand, one goes with (2) and claims that the stimuli are not similar, one needs to accept that, in order for the judgment to shift from anger to fear, almost all the perceptual information conveyed by the target fearful face must be disregarded. But, if this were the case, then the judgment would no longer be perception-based. Moreover, if the evidence is disregarded, nothing prevents additional background information shifting the judgment even further to a radically different emotion, thus generating the problem of how to constrain possible judgments discussed above. Thus, if JS fails to adequately account for the relevant constraints, we need to see whether CP fares any better.

We want to highlight that with CP, we have the possibility of collocating the required constraints at the lower perceptual level of facial-cues. In fact, a straightforward way of accounting for these constraints is to think of them as a range of shared possible values of lower-level facial cues for different emotions. According to this view, in order to explain why anger is recognized in a prototypical fearful face, one needs only suppose that the integration process in the target case highlights the relevance of the shared features. Such features are selected on the basis of background information and expectations, and bound together into an anger-signaling compound. Hence, we have two distinct perceptual compounds, a fear-compound in the case of no conceptual priming, and an anger-compound in the case of conceptual priming. Most importantly, by explaining the difference on the basis of two different compounds, we avoid the dilemma depicted above for the defender of JS. If the integration process is affected before a compound is formed, we can easily understand the possibility that only some relevant perceptual information conveyed by the face is disregarded or given increased saliency. This is precisely what allows two different compounds to be formed. Hence, the recognition process need not disregard the whole information conveyed by the final compound. At the same time, we need not assume that facial expressions for different emotions need to be largely similar. In previous sections, we argued that such compounds are integrated at the level of perception. We therefore hold that different compounds give rise to different experiences,^17^ and that on the basis of these different experiences, two different emotions are recognized.^18^

Hence, CP provides a natural way of explaining why certain recognition outputs are allowed and certain others are not. Which emotion can be recognized in a facial expression depends on the nature, number, and relevance of shared features across different facial expressions and on the integration process. Different outputs of the integration process in turn give rise to different perceptual experiences. Therefore, CP constitutes a better explanation than JS for both the reliability and the (limited) unreliability of emotion recognition across different subjects, insofar as it provides a principled way of constraining the results to be expected. Thus, we conclude that Carroll and Russell (1996) provide a case of CP of perceptual experience, and, more generally, that the perceptual experience of facial expression of emotions is sensitive to background knowledge and expectations. In the next section, we briefly present a recently developed neuro-functional mechanism that supports our view of emotion recognition. If we are correct so far, it seems that CP fares better than JS in accounting for the constraints on possible emotion recognition on the basis of the same stimulus. In addition, we will present further evidence offering independently support for CP over JS. Our strategy is to show that emotion recognition—at least in the case of basic emotions—can be carried out in large part by the perceptual system alone. Therefore, since we presented evidence of particular cases in which background beliefs and knowledge can influence emotion recognition, that influence must be exerted at the level of perception as CP describes, not at the level of post-perceptual cognitive judgments described by JS.^19^

Emotion recognition is a complex process that may involve several perceptual and cognitive mechanisms (see Adolphs, 2002, for an extensive review). However, there is reason to think, at least in the case of basic emotions such as fear, anger, joy, etc., that a large part of the process is carried out by the perceptual system alone. First of all, if an organism’s perceptual system were capable of quickly and automatically processing critical social stimuli and reliably associating these with appropriate behavioral responses and other key features such as non-verbal sounds and lexical labels, this would provide a clear adaptive advantage for the organism. Evidence for this possibility in the case of facial expressions of emotions comes from several sources. One example is research into primates’ facial expressions (Redican, 1982), which shows that in a comparison of new world monkeys (prevalently arboreal) and old world monkeys (prevalently terrestrial), only the latter, which can rely on visual contact with conspecifics, have developed a complex system of facial expressions. This supports both the close connection between facial expressions of emotions and vision and the social value of perceptual integration of facial expressions of emotions (Adams and Kveraga, 2015).

Further interesting evidence for the perceptual nature of emotion recognition comes from computer models (discussed in Adolphs, 2002) designed to achieve comparable performance to humans in evaluating when two facial expressions belong to a different emotional category (even when the structure of the two stimuli is very similar), but that cannot rely on any form of conceptual knowledge about emotions. Moreover, evidence from perceptual priming studies (Carroll and Young, 2005) shows that facilitation effects on emotion recognition are sensitive to the emotional category of the primes (e.g., anger vs. disgust), not only to the positive or negative valence of the emotions. In combination, the evidence discussed here provides support for a quick and reliable perceptual process of emotion recognition that relies on clearly separated perceptual categories that may not always need conceptual knowledge. Hence, if emotion recognition is achieved on the basis of a quick process that relies on discrete perceptual categories, this undermines the claim that cognitive judgment plays a strong role in emotion recognition. Now, if emotions are categorized at the level of perception, shifts in categorization that depend on contextual information (such as those discussed in the previous section) seem to be plausibly explained as special cases, in which background knowledge interferes directly with the perceptual process that leads form feature detection to perceptual categorization, in accordance with CP.

A further consideration in favor of CP is that of explanatory parsimony. If one accepts CP in color perception (Levin and Banaji, 2006), an explanation of the form of CP needs to already be available. Critically, the color case has many relevant similarities with Carroll and Russell (1996). In both studies, target stimuli were of the same broad perceptual kind, namely human faces. In both studies, relevant background knowledge was triggered by conceptual information (a story and a verbal label respectively). However, recall that in Levin and Banaji (2006), subjects were required to perform a perceptual matching task, which rules out the possibility that the influence of racial categories could have been exerted at the level of judgment. Hence, it seems that a CP explanation could account for both cases, whereas JS could account only for the emotion study. If we admit that background knowledge can interfere with the perceptual processing of certain facial features, such as skin color, why should we not favor the same line of explanation (CP) in the case of perceptual processing of other facial features, such as expressions of emotion?^20^

To conclude this section, we wish to examine a final worry based on the claim that the phenomenon described Carroll and Russell (1996) depends on a shift in the subject’s attention, and that it is therefore not a case of CP. This strategy is the one adopted by Pylyshyn to rule out most cases of CP. We need to show that it does not apply in the present case. Pylyshyn (1999) thought that attention shifts exclude CP because the functional role of attention is basically to select (or gate) a subset of the available perceptual information as an input to EV. If this were always the case, a shift in attention would be a pre-perceptual effect amounting to a shift in the input, similar to looking in a different direction in order to gather more information about a stimulus. The resulting perceptual experience would still be different, but it would be causally dependent on such input shift, and this would not be an interesting case of CP. However, we now know that attention shifts can have different effects while the input remains stable.

Here, we have two things to say to counter Pylyshyn’s view. First, it is questionable whether the role that Pylyshyn assigns to attention is the correct or the only possible one. Views of attention differ significantly in terms of the functional role they assign to attention and its underlying processes.^21^ Therefore, it is not so clear that the scope of attentional modulation of perception can be constrained in such a way as to rule out the possibility that attention affects the whole scope of visual processing, including EV. Second, we have seen that if we accept that facial expressions as wholes are perceptually integrated into complex compounds from lower-level facial cues, this must happen after the lower-level cues that constitute such compounds have been processed. Hence, an attentional shift on a facial expression can either affect how the features are integrated, or how the resulting compound is processed. In both cases, it would be an effect that alters perceptual processing itself, not a pre-perceptual effect that changes the input, as Pylyshyn conceived of it. Thus, even if one wishes to call this an attentional shift,^22^ it is nevertheless a shift that happens within perceptual processing, not before. Hence, the case does not meet Pylyshyn’s requirement of attention changing the input to perception. Consequently, it does not undermine CP.^23^

## The Mechanism: Neural Shortcuts, Compound Cues Integration, and Social Vision

So far, we have proposed two reasons for taking the experiment conducted by Carroll and Russell (1996) as evidence for the cognitive penetrability of perceptual experience. The first is that facial expressions of emotion show adaptation, and should therefore be considered as perceptually integrated compounds. The second is that CP is a better explanation for the constrained shifts that can happen in emotion recognition on the basis of background knowledge. However, we have not yet proposed a plausible candidate mechanism that supports such penetration effects.

Before discussing a candidate, we should outline the framework for the search for such a mechanism. It is an open question whether there is only one mechanism that accounts for top-down influences on perceptual integration processes. We have argued elsewhere that we need to distinguish different types of CP (Vetter and Newen, 2014) that may reasonably be assumed to have different underlying mechanisms. We want to describe two routes of top-down influences that are not the preferential candidates for explaining our core example, before outlining a plausible candidate. Top-down influences on perceptual processes may be produced because newly activated beliefs shift our attention and thus relevantly modify the sensory input. Although, as we mentioned above, if attention is conceived differently from Pylyshyn’s account, it may sometimes be a possible mediator of CP, this does not seem to be what happens in the case of contextual background stories (see above). The important candidates as mechanisms of top-down attention modulation are reviewed in Baluch and Itti (2011). A second consideration is that background knowledge is conceptual, and needs to be transformed into a perceptual format before it can causally influence purely perceptual processes. Macpherson (2012) proposes that the top-down modulation of perceptual processes can only be indirect, modulated by activating the relevant imagery. This, however, would only be true if conceptual representations were absolutely separated from imagery and sensory representations. This traditional view of concepts as purely cognitive has been radically called into question by recent data and theories, including embodied concept formation (Barsalou, 1999; Pulvermüller, 2003; Pulvermüller and Fadiga, 2010). Thus, it remains a reasonable option to look for a mechanism that involves direct causal top-down-influences and that may not be purely attentional.

Fortunately for us, there is already a theory available that posits such a top-down mechanism in the case of stimuli that have relevance for social interaction, a paradigmatic class of which is human faces. Moreover, this theory has both a functional component and a neurophysiological model of implementation. The model in question is that of compound social-cues integration (Adams et al., 2010; Adams and Nelson, 2011; Adams and Kveraga, 2015), which relies on the studies of Bar (2003, 2009). According to this view, the anatomy of the visual system supports quick recruitment of higher-level cognitive areas, such as the orbitofrontal cortex (OFC), before a visual stimulus is recognized.^24^ This is possible because the retinal projection of a visual stimulus activates a specific “neural-shortcut,” the magnocellular-pathway (M-pathway), mostly identifiable with the dorsal visual stream.^25^ The M-pathway is known to quickly^26^ project coarse information about the stimulus to the associative areas of OFC. OFC, in turn, presents feedback projections to areas in the ventral stream, including recognition areas in the infero-temporal cortex (IT). Of course, we cannot make inferences from neuroanatomical to functional mechanisms easily. Nevertheless, the existence of many specific and very quick feedback connections in the brain shows at least that nothing in neuroanatomy prevents the occurrence of a process of CP such as the one described above. Moreover, the feedback loop from prefrontal areas (typically associated with reasoning and conceptual knowledge) to visual areas seems to be a plausible preliminary candidate for a neural correlate of CP.

Provided that neuroanatomical characteristics of the brain support the idea of a modulation of perceptual integration exerted by background knowledge, Adams and Kveraga (2015) argue that different social cues, such as gender, age, posture, etc., are relevant to such perceptual integration processes, which they call social vision. In previous sections, we have already provided a sketch of their model, which claims that one of the main tasks of vision is precisely to deliver such integrated meaningful compounds. According to these authors, the plausibility of the idea is supported by evolutionary and everyday considerations about the importance for human beings and other animals of being able to quickly integrate as much socially relevant information as possible. For the purposes of the present paper, however, we need not delve into much detail about the social-vision view. It suffices for our argument that facial-cues, such as eyebrow orientation, mouth shape, gaze direction, and perhaps other facially evident cues such as gender and age, are perceptually integrated together in order to form meaningful emotion-signaling compounds.

If one admits that such integration is possible at the level of the face, then our considerations concerning adaptation and principled constraints on emotion recognition should be enough to show that under certain conditions, the integration process is sensitive to background knowledge, expectations and, possibly, to other high-level cognitive features. We are aware that this is a somewhat unusual way of arguing for CP. We think, however, that perception is a much more dynamic and integrative process that it is described to be in the traditional modular model, and that the evidence we have presented here supports this view. Hence, we conclude that the boundary between perception and cognition should be at least partially blurred.

## Conclusion and Outlook

Cognitive penetration is not only a plausible claim about the perception of objects and physical scenes, but also about the social perception of emotion. The results presented here indicate that we should even go further, and start to investigate the extent to which the perceptual recognition of other social and mental phenomena is shaped by CP. We suggest that face-based recognition of emotion is only one basic component of the most important integration process for humans, namely the integration on the level of person perception (Macrae and Quadflieg, 2010). Person perception is accompanied by an impression formation that should also be explained by a systematic interaction of bottom-up and top-down processes, constituting a person impression (Newen, 2015). Thus, we suggest future work investigating whether CP also holds for the formation of a complex person impression based on perception. One further interesting upshot of this line of investigation is that perceptual processes may essentially rely on the same type of bottom-up and top-down mechanisms, despite the fact that physical objects like trees and social objects like human faces provide us with radically different inputs, and despite the observation that some social stimuli are processed in highly functionally specialized brain areas, like FFA (fusiform face-area) for faces.

### Conflict of Interest Statement

The authors declare that the research was conducted in the absence of any commercial or financial relationships that could be construed as a potential conflict of interest.

## Footnotes

1 This experiment will be of particular importance in the later sections of the paper. Therefore, we present it in somewhat greater detail than the others mentioned.

2 Some of this evidence has been criticized (see, for example Firestone and Scholl, 2014, 2015) and is currently a matter of debate.

3 Block’s case is framed in the context of Burge’s (2010) discussion of perceptual attributives. In this paper, we shall try to phrase the discussion in more general terms.

4 Here, “cognitive” is used in the sense of Block, as concerning conceptual and propositional states.

5 On this topic it is worth noting that on Mroczko-Wąsowicz (2015) construal, “Phenomenal adaptation” is a broader notion that may include non-sensory states. As she points out (p. 2), however, such a notion is quite different from the uncontroversial physiological notion of a perceptual adaptation, which is the one Block employs. We remain neutral with respect to the broader phenomenon of phenomenal adaptation. Nevertheless, following Block, we hold that the more constrained phenomenon of perceptual-adaptation does not involve non-sensory states, which suffices for our argument here.

6 This idea is proposed by Adams and Kveraga (2015), see section below.

7 Carroll and Russell (1996, p. 208).

8 Our notion of Judgment is neutral on how judgments are to be understood. To be clear, we do not think of judgments as necessarily explicit propositional states. Rather, we allow for the possibility of implicit and automatic perceptual judgments.

9 This interaction should work in the same way as in the Butler et al. (2008) case, albeit in the opposite direction. Adaptation and priming can, in some sense, be thought of as two sides of the same coin. As Block points out, the former makes certain things harder to perceptually process, while the latter makes them easier. If we have a perceptual integration process that binds together lower-level features in order to create emotionally meaningful compounds, different factors can make some of these compounds harder or easier to construct, as in, respectively, the adaptation and priming cases. Hence, our account has the advantage of providing a straightforward and unified explanation of both cases.

10 See, for example Froese and Leavens (2014) for a discussion of the interaction between perceptual experience of various physical features (including facial expressions) and conceptual categories from the perspective of the direct perception hypothesis.

11 This argument echoes some of the considerations above concerning perceptual adaptation.

12 By saying that two experiences could be the same or different, we mean that they could be token-experiences of the same type or of a different type.

13 Here, we do not need to take a stance in the debate between dimensionalist views of emotion and views that posit basic emotions. For a theory of emotion that fits nicely with our proposal, see Barlassina and Newen (2014).

14 We do not inquire which specific shifts are allowed and which are not; for the present argument, it is sufficient that emotion recognition changes on the basis of background information do not happen arbitrarily. However, Carroll and Russell (1996) review previous findings (e.g., Tomkins, 1962, 1963) showing that not all background information leads to such a shift. Specifically, the shift does not happen in the case of joy-related information and an anger signaling face (p. 17).

15 We are grateful to Peter Brössel for this example.

16 See Ekman and Friesen (1971).

17 Whether such difference in the experience is best characterized as a difference in content or as a difference in the phenomenal character of the two experiences (or both) is an important open question. However, it goes beyond the scope of the present paper.

18 As Jackendoff (1987) and Prinz (2012) argue, further support for this claim comes from introspection. Introspectively, we have experiences of integrated objects (including faces) and not of unbound low-level features. Therefore, we should situate the locus of conscious perceptual experience after some sort of integration process has taken place, not before.

19 The evidence we present below is in line with a form of direct perception for basic emotions.

20 We know from the previous section that facial expressions are perceptually processed as wholes.

21 See Mole (2011) for a radically different view of attention, and see Mole (2015) and Stokes (2014) for a discussion of attention and its relation to cognitive penetrability.

22 More on this below.

23 We would just like to mention that a CP explanation is consistent with very recent models of emotion recognition and facial expressions such as Carruthers (2015) and Haxby and Gobbini (2011).

24 See also Bar (2003, 2009) and Kveraga et al. (2009, 2011).

25 See Milner and Goodale (1995).

26 As quick as 80 ms.

## References

[B1] Adams R. B. Franklin R. G. Nelson A. J. Stevenson M. T. (2010). “Compound social cues in human face processing,” in The Science of Social Vision, eds Adams R. B. Jr. Ambady N. Nakayama K. Shimojo S. (New York, NY: Oxford University Press), 90–107.

[B2] Adams R. B. Kveraga K. (2015). Social vision: functional forecasting and the integration of compound social cues. Rev. Philos. Psychol. 10.1007/s13164-015-0256-1 [Epub ahead of print]. PMC5726574 29242738

[B3] Adams R. B. Nelson A. J. (2011). “Intersecting identities and expressions: the compound nature of social perception,” in The Handbook of Social Neuroscience, eds Decety J. Cacioppo J. (New York, NY: Oxford University Press), 394–403.

[B4] Adolphs R. (2002). Recognizing emotion from facial expressions: psychological and neurological mechanisms. Behav. Cogn. Neurosci. Rev. 1, 21–62. 10.1177/1534582302001001003 17715585

[B5] Aviezer H. Hassin R. R. Bentin S. Trope Y. (2008). “Putting facial expressions into context,” in First Impressions, eds Ambady N. Skowronski J. (New York, NY: Guilford Press), 255–286.

[B6] Baluch F. Itti L. (2011). Mechanisms of top-down attention. Trends Neurosci. 34, 210–224. 10.1016/j.tins.2011.02.003 21439656

[B7] Bar M. (2003). A cortical mechanism for triggering top-down facilitation in visual object recognition. J. Cogn. Neurosci. 15, 600–609. 10.1162/089892903321662976 12803970

[B8] Bar M. (2009). The proactive brain: memory for predictions. Philos. Trans. R. Soc. Lond. B Biol. Sci. 364, 1235–1243. 10.1098/rstb.2008.0310 19528004 PMC2666710

[B9] Barlassina L. Newen A. (2014). The role of bodily perception in emotion: in defense of an impure somatic theory. Philos. Phenomenol. Res. 89, 637–678. 10.1111/phpr.12041

[B10] Barsalou L. W. (1999). Perceptual symbol system. Behav. Brain Sci. 22, 637–660. 10.1017/S0140525X99532147 11301525

[B11] Bhalla M. Proffitt D. R. (1999). Visual-motor recalibration in geographical slant perception. J. Exp. Psychol. Hum. Percept. Perform. 25, 1076–1096. 10.1037/0096-1523.25.4.1076 10464946

[B12] Block N. (2014). Seeing-as in the light of vision science. Philos. Phenomenol. Res. 89, 560–572. 10.1111/phpr.12135

[B13] Bruner J. S. Goodman C. C. (1947). Value and need as organizing factors in perception. J. Abnorm. Soc. Psychol. 42, 33–44. 10.1037/h0058484 20285707

[B14] Bruner J. S. Postman L. (1949). On the perception of incongruity: a paradigm. J. Pers. 18, 206–223. 10.1111/j.1467-6494.1949.tb01241.x 15409076

[B15] Burge T. (2010). Origins of Objectivity. New York, NY: Oxford University Press.

[B16] Butler A. Oruc I. Fox C. J. Barton J. J. S. (2008). Factors contributing to the adaptation aftereffects of facial expression. Brain Res. 1191, 116–126. 10.1016/j.brainres.2007.10.101 18096142 PMC2538428

[B17] Carroll J. M. Russell J. A. (1996). Do facial expression signal specific emotions? Judging emotions from the face in context. J. Pers. Soc. Psychol. 70, 205–218. 10.1037/0022-3514.70.2.205 8636880

[B18] Carroll N. C. Young A. W. (2005). Priming of emotion recognition. Q. J. Exp. Psychol. 58A, 1173–1197. 10.1080/02724980443000539 16194954

[B19] Carruthers P. (2015). Perceiving mental states. Conscious. Cogn. 10.1016/j.concog.2015.04.009 [Epub ahead of print]. 25935565

[B20] Colombetti G. (2014). The Feeling Body: Affective Science Meets the Enactive Mind. Cambridge, MA: The MIT Press.

[B21] Dunbar R. I. M. (1998). The social brain hypothesis. Evol. Anthropol. 6, 178–190. 10.1002/(SICI)1520-6505(1998)6:5<178::AID-EVAN5>3.0.CO;2-8

[B22] Durgin F. H. Baird J. A. Greenburg M. Russell R. Shaughnessy K. Waymouth S. (2009). Who is being deceived? The experimental demands of wearing a back-pack. Psychon. Bull. Rev. 16, 964–969. 10.3758/PBR.16.5.964 19815806

[B23] Ekman P. Friesen W. V. (1971). Constants across cultures in the face and emotion. J. Pers. Soc. Psychol. 17, 124–129. 10.1037/h0030377 5542557

[B24] Ekman P. Friesen W. V. (1976). Pictures of Facial Affect. Palo Alto, CA: Consulting Psychologists Press.

[B25] Ernst M. O. Bülthoff H. H. (2004). Merging the senses into a robust percept. Trends Cogn. Sci. 8, 162–169. 10.1016/j.tics.2004.02.002 15050512

[B26] Firestone C. Scholl B. J. (2014). Top down effects where none should be found. Psychol. Sci. 25, 38–46. 10.1177/0956797613485092 24297777

[B27] Firestone C. Scholl B. J. (2015). Can you experience top-down effects on perception? Psychon. Bull. Rev. 22, 694–700. 10.3758/s13423-014-0711-5 25520200

[B28] Fodor J. (1984). Observation reconsidered. Philos. Sci. 51, 23–43. 10.1086/289162

[B29] Fodor J. (1988). A reply to churchland’s ‘perceptual plasticity and theoretical neutrality’. Philos. Sci. 55, 188–198. 10.1086/289426

[B30] Froese T. Leavens D. A. (2014). The direct perception hypothesis: perceiving the intention of another’s action hinders its precise imitation. Front. Psychol. 5:65. 10.3389/fpsyg.2014.00065 24600413 PMC3927096

[B31] Gallagher S. (2008). Direct perception in the intersubjective context. Conscious. Cogn. 17, 535–543. 10.1016/j.concog.2008.03.003 18442924

[B32] Hadjikhani N. Kveraga K. Naik P. Ahlfors S. P. (2009). Early (N170) activation of face-specific cortex by face-like objects. Neuroreport 20, 403–407. 10.1097/WNR.0b013e328325a8e1 19218867 PMC2713437

[B33] Hassin R. Aviezer H. Bentin S. (2013). Inherently ambiguous: facial expressions of emotions, in context. Emot. Rev. 5, 60–65. 10.1177/1754073912451331

[B34] Haxby J. V. Gobbini M. I. (2011). “Distributed neural systems for face perception,” in The Oxford Handbook of Face Perception, eds Calder A. J. Rhodes G. Johnson M. H. Haxby J. V. (New York, NY: Oxford University Press), 93–110.

[B35] Haxby J. V. Hoffman E. A. Gobbini M. I. (2000). The distributed human neural system for face perception. Trends Cogn. Sci. 4, 223–233. 10.1016/S1364-6613(00)01482-0 10827445

[B36] Jackendoff R. (1987). Consciousness and the Computational Mind. Cambridge: The MIT Press.

[B37] Keltner D. Ekman P. Gonzaga G. C. Beer J. (2003). “Facial expression of emotion,” in Handbook of Affective Sciences, eds Davidson R. J. Scherer K. R. Goldsmith H. H. (New York, NY: Oxford University Press), 415–431.

[B38] Krueger J. (2012). Seeing mind in action. Phenomenol. Cogn. Sci. 11, 149–173. 10.1007/s11097-011-9226-y

[B39] Kveraga K. Boshyan J. Bar M. (2009). “The proactive brain: using memory-based predictions in visual recognition,” in Object Categorization: Computer and Human Vision Perspectives, eds Dickinson S. Tarr M. Leonardis A. Schiele B. (Cambridge: Cambridge University Press), 384–400.

[B40] Kveraga K. Ghuman A. S. Kassam K. S. Aminoff E. Hamalainen M. S. Chaumon M. (2011). Early onset of neural synchronization in the contextual associations network. Proc. Natl. Acad. Sci. U.S.A. 108, 3389–3394. 10.1073/pnas.1013760108 21300869 PMC3044398

[B41] Lee T. S. Nguyen M. (2001). Dynamics of subjective contour formation in the early visual cortex. Proc. Natl. Acad. Sci. U.S.A. 98, 1907–1911. 10.1073/pnas.98.4.1907 11172049 PMC29355

[B42] Levin D. Banaji M. (2006). Distortions in the perceived lightness of faces: the role of race categories. J. Exp. Psychol. Gen. 135, 501–512. 10.1037/0096-3445.135.4.501 17087569

[B43] Lupyan G. (2012). Linguistically modulated perception and cognition: the label-feedback hypothesis. Front. Psychol. 3:54. 10.3389/fpsyg.2012.00054 22408629 PMC3297074

[B44] Lupyan G. (2015). “Cognitive penetrability of perception in the age of prediction,” in Cognitive Penetration of Perception, Review of Philosophy and Psychology, eds Siegel S. Jenkins Z. (forthcoming).

[B45] MacCauley R. N. Henrich J. (2006). Susceptibility to the Müller-Lyer illusion, theory neutral observation, and the diachronic cognitive penetrability of the visual input system. Philos. Psychol. 19, 79–101. 10.1080/09515080500462347

[B46] Macpherson F. (2012). Cognitive penetration of colour experience. Rethinking the issue in light of an indirect mechanism. Philos. Phenomenol. Res. 84, 24–62. 10.1111/j.1933-1592.2010.00481.x

[B47] Macrae C. N. Quadflieg S. (2010). “Perceiving people,” in Handbook of Social Psychology, 5th Edn, Vol. 1, eds Fiske S. T. Gilbert D. T. Lindzey G. (Hoboken, NJ: John Wiley & Sons), 428–463.

[B48] Milner A. D. Goodale M. A. (1995). The Visual Brain in Action. New York, NY: Oxford University Press.

[B49] Mo L. Xui G. Kay P. Tan L. H. (2011). Evidence for the left-lateralized effect of language on preattentive categorical perception of color. Proc. Natl. Acad. Sci. U.S.A. 108, 14026–14030. 10.1073/pnas.1111860108 21844340 PMC3161602

[B50] Mole C. (2011). Attention is Cognitive Unison. New York, NY: Oxford University Press.

[B51] Mole C. (2015). “Attention and cognitive penetration,” in The Cognitive Penetrability of Perception, eds Raftopoulos A. Zeimbekis J. (New York, NY: Oxford University Press), 218–238.

[B52] Mroczko-Wąsowicz A. (2015). “What can sensorimotor enactivism learn from studies on phenomenal adaptation in atypical perceptual conditions? A commentary on rick grush and colleagues,” in Open MIND: 16, eds Metzinger T. Windt J. M. (Frankfurt am Main: MIND Group), 1–17. 10.15502/9783958570283

[B53] Newen A. (2015). “Understanding others—the person model theory,” in Open MIND: 26, eds Metzinger T. Windt J. M. (Frankfurt am Main: MIND Group), 1–28. 10.15502/9783958570320

[B54] Newen A. Welpinghus A. Juckel G. (2015). Emotion recognition as pattern recognition. The relevance of perception. Mind Lang. 30, 187–208. 10.1111/mila.12077

[B55] Pessoa L. (2013). The Cognitive-Emotional Brain: From Interactions to Integration. Cambridge, MA: The MIT Press.

[B56] Prinz J. (2012). The Conscious Brain: How Attention Engerders Experience. New York, NY: Oxford University Press.

[B57] Pulvermüller F. (2003). The Neuroscience of Language: On Brain Circuits of Words and Serial Order. Cambridge: Cambridge University Press.

[B58] Pulvermüller F. Fadiga L. (2010). Active perception: sensorimotor circuits as a cortical basis for language. Nat. Rev. Neurosci. 11, 351–360. 10.1038/nrn2811 20383203

[B59] Pylyshyn Z. W. (1984). Computation and Cognition: Toward a Foundation for Cognitive Science. Cambridge, MA: The MIT Press.

[B60] Pylyshyn Z. W. (1999). Is vision continuous with cognition? The case for cognitive impenetrability of visual perception. Behav. Brain Sci. 22, 341–365. 10.1017/S0140525X99002022 11301517

[B61] Raftopoulos A. (2014). The cognitive impenetrability of the content of early vision is a necessary and sufficient condition for purely nonconceptual content. Philos. Psychol. 27, 601–620. 10.1080/09515089.2012.729486

[B62] Redican W. K. (1982). “An evolutionary perspective on human facial displays,” in Emotion in the Human Face, 2nd Edn, ed. Ekman P. (Cambridge, MA: Cambridge University Press), 212–280.

[B63] Salin P. A. Bullier J. (1995). Corticocortical connections in the visual system: structure and function. Physiol. Rev. 75, 107–154. 7831395 10.1152/physrev.1995.75.1.107

[B64] Schwiedrzik C. M. Ruff C. C. Lazar A. A. Leitner F. C. Singer W. Melloni L. (2014). Untangling perceptual memory: hysteresis and adaptation map into separate cortical networks. Cereb. Cortex 24, 1152–1164. 10.1093/cercor/bhs396 23236204 PMC3977616

[B65] Stefanucci J. K. Geuss M. N. (2009). Big people, little world: the body influences size perception. Perception 38, 1782–1795. 10.1068/p6437 20192128 PMC3298356

[B66] Stokes D. (2013). Cognitive penetrability of perception. Philos. Comp. 8, 646–663. 10.1111/phc3.12043

[B67] Stokes D. (2014). Cognitive penetration and the perception of art. Dialectica 68, 1–34. 10.1111/1746-8361.12049

[B68] Stokes D. Bergeron V. (2015). Modular architectures and informational encapsulation: a dilemma. Eur. J. Philos. Sci. 10.1007/s13194-015-0107-z [Epub ahead of print].

[B69] Stout R. (2012). What someone’s behavior must be like if we are to be aware of their emotions in it. Phenomenol. Cogn. Sci. 11, 135–148. 10.1007/s11097-011-9224-0

[B70] Tomkins S. S. (1962). Affect, Imagery, Consciousness, Vol. 1. London: Springer.

[B71] Tomkins S. S. (1963). Affect, Imagery, Consciousness, Vol. 2. London: Springer.

[B72] Vetter P. Newen A. (2014). Varieties of cognitive penetration in visual perception. Conscious. Cogn. 27, 62–75. 10.1016/j.concog.2014.04.007 24836978

[B73] Winawer J. Witthoft N. Frank M. C. Wu L. Wade A. R. Boroditsky L. (2007). Russian blues reveal effects of language on color discrimination. Proc. Natl. Acad. Sci. U.S.A. 104, 7780–7785. 10.1073/pnas.0701644104 17470790 PMC1876524

[B74] Witzel C. Valkova H. Hansen T. Gegenfurthner K. (2011). Object knowledge modulates colour appearance. Iperception 2, 13–49. 10.1068/i0396 23145224 PMC3485772

[B75] Zahavi D. (2011). Empathy and direct social perception: a phenomenological proposal. Rev. Philos. Psychol. 2, 541–558. 10.1007/s13164-011-0070-3

